# Induction of bone formation in abdominal implants constituted by collagen sponges embedded with plant-based human transforming growth factor family proteins in ectopic dog model

**DOI:** 10.1186/s40634-014-0011-z

**Published:** 2014-07-16

**Authors:** Juan Carlos Jacinto-Tinajero, Daniel Ascencio, Brenda Marquina, Jorge Barrios-Payán, Maria Concepcion Gutierrez, Miguel Gomez Lim, Rogelio Hernández Pando

**Affiliations:** Experimental Pathology and Surgery Departments, National Institute of Medical Sciences and Nutrition “Salvador Zubiran”, Mexico city, Mexico; Plastic Surgery, Angeles Hospital, Mexico city, Mexico; Experimental Biology Department, Metropolitan University, Mexico city, Mexico; Department of Genetic Engineering in Plants, National Politechnique Institute Center of Research and Advanced Studies, Irapuato, Mexico; Department of Pathology, National Institute of Medical Sciences and Nutrition ‘Salvador Zubirán’, Section of Experimental Pathology, Mexico city, DF 14000 Mexico

**Keywords:** Heterotopic bone, Tissue engineering, Bone morphogenetic proteins

## Abstract

**Background:**

Trauma, osteomyelitis, bone tumour resections and congenital deformities are the main causes of bone deficiency in which autologous graft is the preferred treatment, but usually the bone supplies are limited.

**Methods:**

An experimental model of heterotopic bone formation in the subcutaneous abdominal area of dogs was developed. This model consists in omentum wrapped implants constituted by collagen type 1 sponges embedded with demineralized bone powder, calcium cloride, thrombin and platelet rich plasma; the implant is totally converted in trabecular bone after four months of implantation. This model was improved by accelerating bone production, after the isolation of the most conspicuous histological constituents (inflammatory, bone and adipose tissues) by laser microdisection and purified from them RNA that was used to determine by RT-PCR the gene expression kinetics of the most important growth bone factors. Then, the most abundant and rapidly synthesized factors were produced by genetic engineering in tobacco plants.

**Results:**

Bone morphogenetic proteins 2 and 7 and transforming growth factor-β1were the most rapidly and highly synthesized factors, and they were efficiently produced in a genetic engineering plant based system in tobacco leaves. Their incorporation as recombinant proteins in the scaffold collagen sponge induced in just one month mature heterotopic bone.

**Conclusion:**

This study demonstrates for the first time that this plant system is able to produce recombinant bone growth factors in high amount and at low cost, and they were highly efficient to rapidly induce bone formation in abdominal implants potentially useful for autotransplantation.

## Background

There are several problems that cause bone defects; the most important are trauma, osteomyelitis, bone tumour resections and development deformities [1]. In cases in which bone reconstruction is associated with bone defect, the traditional therapies are usually not successful and some cases require amputation [2]. Due to these problems the treatment of bone defect has been recently studied, being vascularized bone grafting and distraction osteogenesis the election therapies [3]. Another new perspective in the treatment of this problem is tissue engineering.

Bone tissue engineering is based in part of tissue induction, that can be defined as a process in which a tissue or product derived from it causes a second undifferentiated tissue to differentiate into bone. This process is orchestrated by signaling molecules that govern cell proliferation, migration, and differentiation, which depend on the microenvironment and recruitment of progenitor cells, recapitulating embryonic development [4]. Indeed, this process is similar to heterotopic bone formation [5] which usually starts with the proliferation of mesenchymal and perivascular undifferentiated cells, followed by their osteoblastic cell differentiation producing mature bone tissue. Thus, bone induction requires at least of three elements: osteoprogenitor cells, osteoinductive factors and supporting extracellular matrix [6,7]. We developed an experimental model of heterotopic bone formation by the integration of these three elements in the subcutaneous abdominal area of dogs [8]. This model consist in omentum wrapped implants constituted by collagen type I sponges, which is the supportive matrix that have the osteo-inductive factors bone morphogenetic proteins (BMP); platelets rich plasma that is another source of BMP and other growth factors such as transforming growth factor beta I (TGFβ1), platelet derived growth factor (PDGF) and insulin like growth factor I and II (IGF). We also added to the implants thrombin that induces the production of fibrinogen and fibrin which are also osteo-inductive factors and promote vascular proliferation after binding vascular endothelial growth factor (VEGF); moreover, both proteins give a soft consistency and moldable condition permitting to give the implant the desired shape and size that is maintained by the neoformed bone. Although this is an efficient model of heterotopic bone formation, it needs a long time for complete bone formation (four months). The aim of the present work was to accelerate a more efficient bone production in this model. In order to do this, we first isolated using laser microdisection the most conspicuous histological constituents and purified from them total RNA, that was used to determine by RT-PCR the gene expression kinetics of the most important growth bone factors. In a second part of the study, the most abundant and early expressed growth factors (BMP-2, BMP-7, TGF-β1) were produced in *Nicotiana benthamiana* as recombinant proteins and added to the implants. We decided to use plants for expressing these factors because plants do not contain animal pathogens, have low production costs and rapid production of recombinant proteins is easily performed [9,10]. We used a transient expression system which does not result in transgenic plants. Our results showed a much more rapid production of heterotopic bone (1 month), which could be useful for auto-transplantation.

## Methods

### Experimental model of heterotopic bone formation

Fourteen mongrel dogs, 4–6 months old and weighing 15-20 kg, free of evident infectious or parasitic illnesses, were anesthetized with pentobarbital (16 mg/kg) and atropine (0.5 mg/kg). Anesthesia was maintained with 2% Ethrane (enflurane). An abdominal midline incision was performed and the great omentum was dissected tailoring a flap with a pedicle from the right gastro-omental artery, keeping the omentum flap immersed in the subcutaneous tissue. The implant was constituted by a sponge of type I collagen and polyvinylpyrrolidone (Fibroquel; Aspid Laboratory, Mexico), embedded with 5 ml of demineralized bone powder (DBP) (Veterinary Transplant Services, Seattle, WA), 5 ml of dog platelet-rich plasma, 10,000 IU of bovine thrombin (Behring, Mexico), and 2.5 ml of 10% calcium cloride (14.7 mg). The mixture of platelet-rich plasma, DBP, thrombin, and collagen formed a soft material that after 1 or 2 minutes was easily moldable and allowed the formation of a cylinder that was wrapped with the omentum flap. Animals were maintained in conventional room with food and water ad libitum. This protocol was approved by the Animal Research and Ethics Committees of the National Institute of Medical Sciences and Nutrition, according to Mexican and International laws.

### Kinetics of growth factors genetic expression from specific histologic compartments

The mosaic expression of growth factors during skeletogenesis indicates that diverse bones have different expression of them and it should have therapeutic significance [5]. We hypothesized that a similar situation should exist during heterotopic bone formation. Thus, abdominal implants were dissected and removed after 7, 14, 28, 60, 90, and 120 days of their implantation. Each implant was divided into quadrants and fixed by immersion in 4% paraformaldehyde diluted in phosphate buffered saline solution. After 3 days of decalcification with formic acid, the tissue was embedded in paraffin, sectioned, and stained with hematoxylin and eosin. At least two sections were obtained from each quadrant. In these slides, the most conspicuous tissues were the connective-inflammatory tissue, adipose tissue, and osteoid or mature bone trabeculae. We laser captured these areas by separate under direct microscopic visualization by melting of the selected regions onto a thermoplastic film mounted on optically transparent LCM caps using the Arcturus XP equipment (Arcturus Engineering, Mountain View, CA, USA). The thermoplastic film containing the microdissected tissue cells was used to isolate total RNA using the RNeasy mini kit (Qiagen, Valencia, CA, USA). Quality and quantity of RNA were evaluated through spectrophotometry (260/280 nm) on agarose gels. Reverse transcription of the mRNA was performed using 5 μg RNA, oligo-dT and the Omniscript kit (Qiagen). Real-time PCR was performed using the 7500 real-time PCR system (Applied Biosystems, Foster City, CA, USA) and Quantitect SYBR Green Master-mix kit (Qiagen). Standard curves of quantified and diluted PCR product, as well as negative controls, were included in each PCR run. Specific primers were designed using the program Primer Express (Applied Biosystems) for the following targets: TGF-β1 F: ATG TCA CTG GAG TCG TGA GGC. TGF-β1 R: CCT CGA CTT CCC CTC CAT G. BMP 2 F: AGC CTG GCC AAC ACC GT. BMP 2 R: CAA AGA TTC TAA TTC TTC GT. BMP 6 F: GCG CCT CAG CCC CAA. BMP 6 R: AGG AGT TCT TCC TCT CTC TA. BMP 7 F: GCA GGA CTT GAT CAT CGC TC. BMP 7 R: CCC TCA CAG TAG TAA GCG CGA. IGF 1 F: CAA TTG CTG TTG GGT CGT CA. IGF 1 R: CGC TGA ACA GGG CTT CTG TA. IGF 2 F: GGA CAG CCT GCT TGC TCA AT. IGF 2 R: TTC ACT TGC TCG CAG TTT TCC HPRT F: GGA CAG TAG GAC TGA GCG GCT. HPRT R: CTA CGA TGT GAT GGC CTC CC.

### Production of recombinant growth factors in Nicotiana benthamiana

As shown below our study of gene expression kinetics of growth factors showed that BMP-2 was the most rapid and highest expressed factor, followed by BMP-7 and TGF-β1. Thus, we produced these as recombinant proteins in plants.

The sequences employed (TGFβ1 GenBank: X02812.1 TGFβ1, BMP2 NM_001200.2, BMP7 NM_001719.2) were optimized for expression in plants and synthesized by Genscript.com for cloning. All sequences contained a 6xHis tag at the 3′end and were flanked with BsaI sites and cloned in the pICH31070 vector. Orientation and codon in-frame for all constructs were confirmed by restriction analysis and sequencing. All expression vectors were kindly provided by Dr. Yuri Gleba (Icon Genetics) [11]. The expression vectors were introduced into Agrobacterium tumefaciens GV3101 by electroporation. The infiltration of Agrobacterium into N. benthamiana plants was performed as described [12]. In brief, Agrobacterium overnight cultures were grown in YENB medium to high cell density (OD600 = 0.6) and were diluted into infiltration buffer (10 mM MES, pH 5.5; 10 mM MgSO4) to reach the desired concentration. Bacterial suspensions were infiltrated into plants leaves using vacuum. After infiltration, the plants were grown under glasshouse conditions for 10 days. Leaves were then harvested, frozen in liquid nitrogen and stored at 80ºC. Subsequently they were macerated and resuspended in extraction buffer (10 mM PBS pH 7.4, 5 M NaCl, 0.5 M EDTA pH 8.0, 0.01% Triton X-100, 50 mM sodium ascorbate, 100 mM PMSF, 100 mM DTT) at 1:2 (v/w) ratio and centrifuged (8000 × g) for 20 min at 4ºC. The supernatant was retained and the concentration of total soluble protein (TSP) was determined by the Bradford method (Bio-Rad) with BSA as standard. For purification of the growth factors, the extracts were loaded on a HisTrap HP column (GE Healthcare Life Sciences), the column washed and the 6xHis tag-containing proteins eluted as described by the manufacturers. Fractions were collected and analyzed by SDS-PAGE. Those fractions showing the factors in the purest form were pooled and stored. The identity of each factor was confirmed by western-blot. Briefly, equal amounts of protein from each factor were separated on a non-reducing 12% SDS-PAGE, transferred to nitrocellulose membrane and blocked with 5% fat free milk in TBS/0.05% Tween 20 for 2 h. The membrane was incubated with anti-TGFβ1 (Santa Cruz Biotechnology), anti-BMP-2 or BMP-7 (Peprotech) polyclonal rabbit antibodies at 4°C overnight. After washing, the membrane was incubated with HRP-conjugated anti-rabbit antibody (Santa Cruz Biotechnology) in Tris-Tween 20 for 1 h. Following several washes, the blot was developed by chemiluminescence (Super Signal West Pico Chemiluminescent Substrate, Thermo Scientific). Control blots were processed without incubation of the primary Ab.

### Effect of recombinant BMP2, BMP7 and TGFβ1 in the heterotopic bone production in abdominal implants

In two independent experiments, eight mongrel dogs were used for the evaluation of heterotopic bone formation in implants with recombinant growth factors. Following the same procedure described above, in the right side were made two implants, in the upper implant was added 2 cc (200 mg) of recombinant BMP-2, in the lower implant was added the same amount of BMP-7, while in the left side three implants were produced, in the top implant was added the same concentration of TGF-β1, in the middle implant was added the three growth factors in the same doses and the bottom implant was the control without recombinant factors. Right and left implants were removed after 1 and 4 weeks and evaluated histologically for bone formation in sections stained with hematoxilin and eosin. The percentage of the tissue implant conformed by trabecular bone was determined by automated morphometry. Other sections were stained with Masson trichrome and von Kossa techniques in order to evaluate bone maturation. Cellular proliferation was evaluated by the detection of proliferative cell nuclear antigen (PCNA) using conventional immunohistochemistry [8].

### Statistical analysis

Student’s T-test was used to determine statistical significance of bone growth factors expression and histomorphometry. p < 0.05 was considered significant.

## Results

### Gene expression kinetics of growth factors during heterotopic bone formation

**Figure 1:**
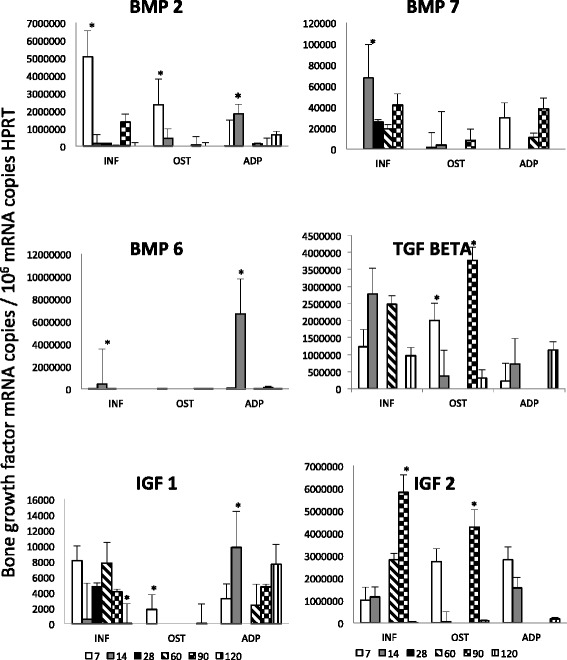
**Kinetics of bone growth factors gene expression during heterotopic bone formation.** Bone trabeculae (OST), inflammatory tissue (INF) and adipose tissue (ADP) were isolated from the implants using laser microdisection and used to isolate total RNA to determine by RT-PCR the indicated bone growth factor during the heterotopic bone formation. Numbers indicate the day when the implant was obtained after the implantation. Results are present as mean and standard deviation from three dogs in the indicated time points. Two independent experiments were run with similar results. Asterisks represent statistical significance comparing the groups in each time point (p < 0.05).

Figure 1 shows that at 7 days of implantation, BMP-2 was the highest expressed factor in the connective inflammatory tissue around or distant from small areas of immature bone, in which also was determined a high expression of this growth factor, while in the adipose tissue the expression of BMP-2 raised its peak after two weeks of implantation being the number of transcripts similar than in the osteoid after one week of implantation. Then, the expression of BMP-2 progressively declined in the three different tissues. TGF-β1 was also highly expressed after one week of implantation in immature bone, and this expression was twofold higher after two months when well formed trabecular bone was already produced, while in the connective tissue around bone trabeculae the peak of TGF-β expression was after 2 and 4 weeks of implantation and similarly expressed after 4 months in the three different tissues (Figure 1).

The kinetics of BMP-7 expression was different; this factor showed its highest expression after 2 weeks of implantation in the inflammatory cells located around or distant to bone trabeculae, followed by temporal decrease and increasing again after 60 days showing similar level of expression in the adipose tissue, while a mild and stable expression was detected in the trabecular bone. In contrast, BMP-6 was only expressed after one week of implantation in the inflammatory and adipose tissue (Figure 1). IGF-2 exhibited its highest level after 3 months of implantation in the inflammatory and bone tissue, while in the adipose tissue it raised its peak after one week of implantation. In contrast, IGF-1 exhibited a lower and constant expression (Figure 1).

### Production of recombinant BMP-2, BMP-7 and TGF-β1 in tobacco and their effect in heterotopic bone formation

**Figure 2:**
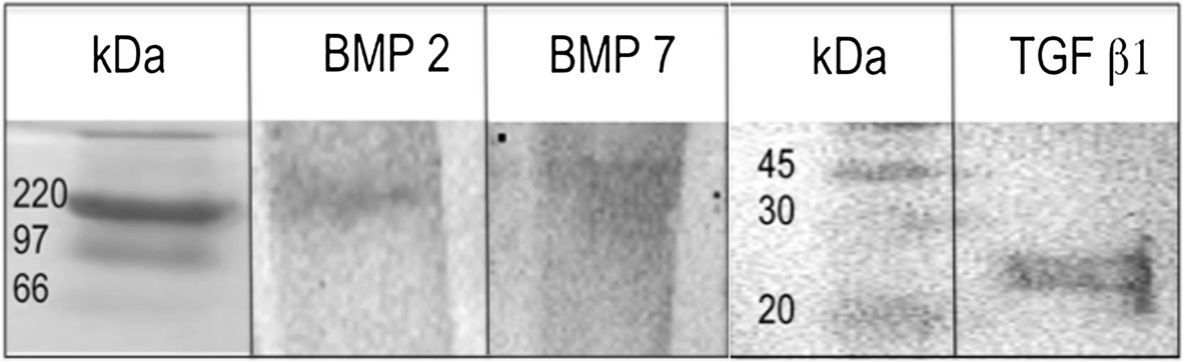
**Identification by western-blot of recombinant BMP-2, BMP-7 and TGF-β1 from** ***Nicotiana benthamiana*** **leaves.** Crude extracts from plants containing each factor and obtained as described in the text were filtered on 0.2 μm membranes and loaded on a HisTrap HP column. Each factor was loaded separately. Fractions were dialyzed against PBS, loaded on a SDS-PAGE gel, transferred to nitrocellulose membranes and incubated with specific rabbit policlonal antibodies to confirm its identity. Molecular weight markers (kDa) are indicated on the left.

**Figure 3:**
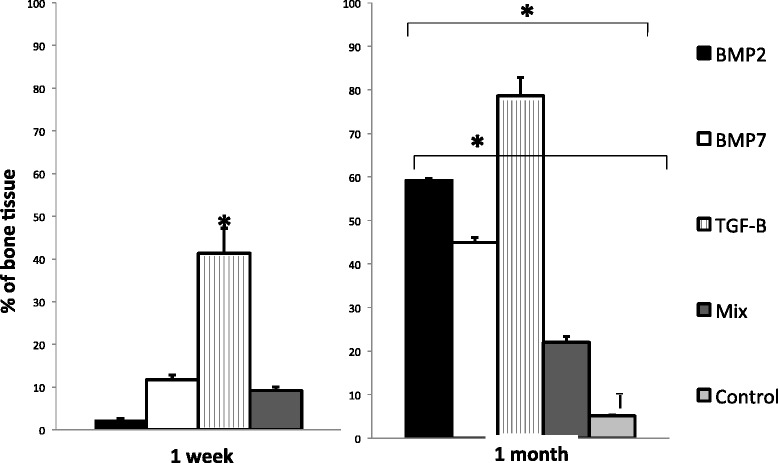
**Determination of the percentage of bone tissue by automated morphometry after one and four weeks of implantation adding the indicated growth factors.** Results are presented as means and standard deviation of three different dogs in the indicated time points. Mix means implants with the three factors in the same concentration. Asterisk represent statistical difference (p < 0.005) among the indicated groups. Two independent experiments were run with similar results.

**Figure 4:**
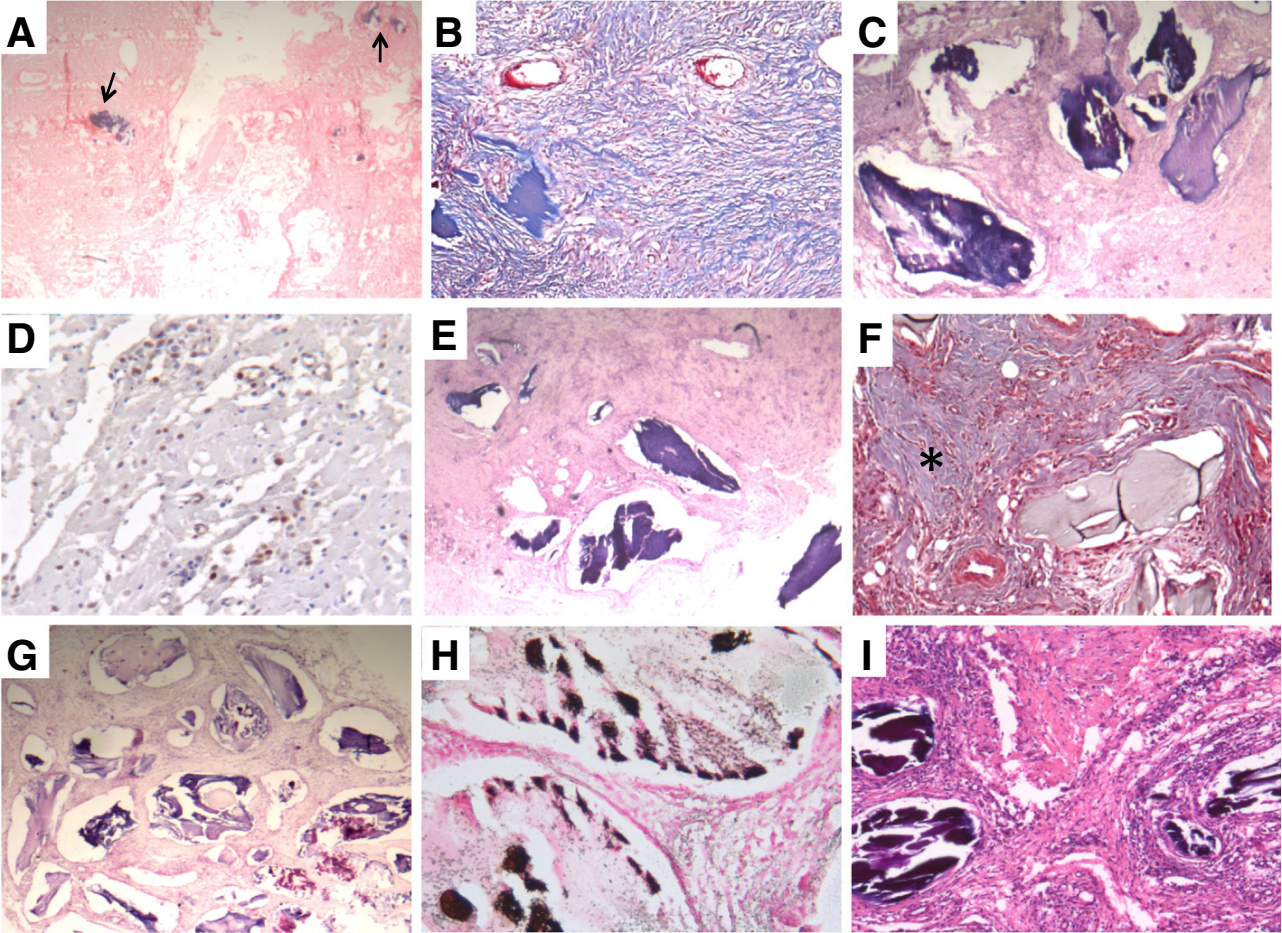
**Representative histology of implants after 4 weeks of abdominal implantation.** **A)** Control implant without any factor is essentially constituted of fibroadipose tissue with small areas of bone (arrows). **B)** Fibrous tissue in control implant is well demonstrated by Masson trichromic staining. **C)** In contrast, implant with recombinant BMP-2 shows large mature bone trabeculae surrounded by fibrous or mesenchymal tissue. **D)** This mesenchymal tissue shows numerous cells with PCNA positive immunostaining. **E)** Similar bone production is observed in implant with BMP-7. **F)** This implant with BMP7 shows extensive areas of osteoid in section stained with Masson technique (asterisk). **G)** Even more bone trabeculae are seen in an implant with addition of TGF-1. **H)** They are mature trabeculae constituted by well calcified bone as show by Von Kossa staining. **I)** In comparison, fewer bone trabeculae were induced in implant with all the TGF family factors.

BMP-2, BMP-7 and TGF-β1 were successfully produced in tobacco leaves and their molecular identity was confirmed by western-blotting (Figure 2). After purification, the same amount of each growth factor was added to the implants and the bone production was evaluated after 1 and 4 weeks. All animals survived the surgical procedure. Neither infection nor significant local inflammation was seen. During the surgical resection, the implants showed excellent vascularization without excessive adherences, allowing for an easy surgical extraction and maintained their cylindrical shape. Control implants that did not have the recombinant proteins showed scarce bone formation, less than 5% after one month of implantation (Figures 3 and 4). In contrast, the implants that had recombinant growth factors showed trabecular mature bone since the first week of implantation, being 3%, 12% and 40% in implants with recombinant BMP-2, BMP-7 and TGF-β1 respectively. The percentage of bone formation progressively increased, being more than 80% after one month of implantation with TGF-β1, followed by 60% and 45% in the implants with BMP-2 and BMP-7 respectively. Both factors induced the nuclear expression of PCNA in the mesenchimal cells around the bone trabeculae, and particularly BMP7 induced relatively large areas of osteoid material (Figure 4). Thus, cellular proliferation and differentiation are involved in heterotopic bone production. The addition of the three growth factors in the same implant showed lesser production of heterotopic bone than the implants that received each factor independently, histologically this implants showed numerous small blood vessels and osteoid. This efficient and relatively rapid bone production confirms the effective biological efficiency of these factors produced in plants.

## Discussion

The preferred treatment of bone deficiency is autologous graft with bone harvested from sites such as the iliac crest [13], but usually the available autologous bone supplies are limited and harvesting is painful and with risk of infection. Bone allograft is an alternative, but it has limited success because the delayed time to fusion or incomplete graft incorporation due to the elicited immune response [14]. Thus, several novel approaches are currently being explored, such as tissue engineering [7].

Well-vascularised intramuscular sites are highly favorable to the induction of bone formation and this has motivated the manufacturing of heterotopic bone for autologous transplantation [15]. We used another strategy for heterotopic bone formation, producing abdominal implants constituted by a type I collagen sponge embedded with DBP, platelets and calcium cloride wrapped with omentum [8]. We now improved this system by the addition of recombinant BMP-2, BMP-7 or TGF-β1which were the quickest and highest bone growth factors produced in this model.

The induction of bone tissue requires osteoprogenitor cells, osteoinductive factors and a supporting extracellular matrix that must interact in a highly regulated process [5–7]. We wrapped the implants with omentum, which is constituted by adipose cells, fibrous tissue and a blood vessels network. Adipose tissue is a mesodermally derived organ that has a stromal population containing endothelial, smooth muscle and stem cells [16]. Adipose tissue-derived stem cells share many characteristics of its counterpart in bone marrow including multilineage differentiation [17]. Thus, the omentum adipose tissue is a good source of mesenchymal stem cells for heterotopic bone formation.

Adequate vascularization is another important attribute for heterotopic bone production [18], and the omentum has an extensive blood vessels network that is a good source of nutrients, oxygen, and growth factors, creating a proper microenvironment for bone induction. We added platelet rich plasma to our implants because these cells are a source of growth factors that induce blood vessels production [19]. High vascularization is also related with an adequate inflammatory response with numerous macrophages that induce chemotaxis of mesenchymal stem cells and promote their differentiation to bone cells through production of osteogenic factors [8]. This was clearly demonstrated by our gene expression kinetics study which showed the inflammatory tissue as the site of the highest expression of BMP and TGF-β1.

A significant focus of research on tissue engineering has been the developing of ideal carriers for bone growth factors [20]. The carrier should act as three-dimensional space scaffolding across which de novo bone formation can occur; and also should maintain an effective concentration of growth factors and containing them in order to avoid extraneous bone formation. Collagen is considered a good carrier, because it is a good source of adhesion-signaling molecules and is also a support for blood vessels and parenchymal cells. We used a type I collagen sponge as carrier, because it is also osteoinductive and favored vascular growth due to its physical characteristics. There is also a good amount of fibrous tissue in the omentum, which contributed to the confinement of the implant and growth factors, avoiding the bone production outside the implant and facilitates its surgical resection avoiding the formation of adherences.

Other essential elements in bone formation are specific growth factors, such as BMP, FGF, PDGF, IGFs [21,22]. BMP is the largest sub group of growth factors that belong to the TGF-β superfamily; they are pleiotropic regulators that mediate various sequential cellular responses such as: chemotaxis and proliferation of progenitor cells, differentiation into osteoblast, vascular invasion, bone formation, remodeling and bone marrow differentiation [6,21]. For bone induction, the most commonly utilized BMPs are BMP2 and 7. BMP2 acts upstream inducing global cellular mobilization (day 1 to 3), whereas BMP7 acts on bone differentiation (day 2 to 5) [2]. These kinetics patterns were also observed in our model, during the first week of implantation BMP2 was highly expressed, while BMP7 raised its maximal expression during the second week. We produced both factors in tobacco leaves as recombinant proteins and added into the implants. After one month, 60% and 45% of the implant was constituted by trabecular bone when added BMP-2 or BMP-7 respectively. The induction of cell proliferation by both BMPs was demonstrated by the immunohistochemistry detection of the cell proliferation marker PCNA, which showed numerous positive cells in the mesenchymal tissue around bone trabeculae. Thus both recombinant proteins are efficient inducing a more rapid bone production in this model. Indeed, both BMPs are now available in the clinical setting [2], and recent evidence has shown that heterodimeric BMPs may have a greater effect than homodimers alone [23]. However in our experimental conditions, we did not observe this better effect when both factors were added in the same implant at the same amount; perhaps because the dose, proportions and the timing for their administration. More investigation should be done exploring this important issue, which we consider an easy procedure considering that the implant is below the abdominal skin facilitating the direct BMP administration by injection.

The most efficient factor to induce bone formation in our model was TGF-β1. This cytokine increases bone formation mainly by recruiting osteoblast progenitors and stimulating their proliferation, as well as by promoting the early stages of bone differentiation [24]. Results from *P. ursinus* have shown that low doses of TGF-β1 combined with recombinant human osteogenic protein-1 result in the induction of massive ossicles in heterotopic sites (rectus abdominis muscle) as early as 15 days [25]. In our model, 80% of the abdominal implants were constituted by trabecular bone with only one dose of TGF-β1 administered alone. However, there are some potential disadvantages of this procedure. First, TGF-β1 not only modulates bone formation but can also stimulate osteoclast formation, which can be prevented by its administration during early implantation. Second, the half-life of TGF-β1 is short (2 min), implying the need for a matrix to allow for its slow release, we believe that collagen matrix can do this function, as well as containing it avoiding other activities of TGF-β outside the bone.

Although both BMP-2 and BMP-7 are potent osteoinductive agents, very large doses are required to produce an adequate biologic response. Indeed, the concentration of BMPs used in clinical trials are more than one million times greater than that found in human bone [26], and recombinant BMP is quite expensive. In this sense, plants represent an ideal alternative for production of recombinant proteins as they lack mammalian pathogens and the proteins produced are identical to the native ones and highly cost effective [9,10,27].

## Conclusion

Implants constituted by collagen type 1 sponge embedded with DBP, calcium cloride, thrombin, platelets and recombinant TGFβ1 wrapped with omentum efficiently induce heterotopic bone formation in the subcutaneous abdominal area in dogs. It was demonstrated for the first time that growth factors produced in plants in high amounts and at low cost were highly efficient to rapidly induce heterotopic bone formation potentially useful for autotransplantation.
